# Applications of Genetic Algorithms for Designing Efficient Parking Shelters with Conoid-Shaped Roofs

**DOI:** 10.3390/ma18133083

**Published:** 2025-06-29

**Authors:** Jolanta Dzwierzynska, Anna Szewczyk, Ewelina Gotkowska

**Affiliations:** Faculty of Civil and Environmental Engineering and Architecture, Rzeszow University of Technology, Al. Powstancow Warszawy 12, 35-959 Rzeszow, Poland; a.szewczyk@prz.edu.pl (A.S.); e.gotkowska@prz.edu.pl (E.G.)

**Keywords:** steel structures, finite element method, parametric design, generative design, optimization, genetic algorithms, dynamic analysis, vehicle impact, parking shelter

## Abstract

Rapid urbanization, excessive motorization, and the imperative to reduce carbon footprints are driving the search for sustainable urban space solutions. One promising approach involves the effective design of small-scale architecture, such as parking shelters, optimized for structural material consumption and resilience to vehicle impacts. This research employed a novel approach during the initial design phase. Genetic algorithms and optimization techniques were utilized to define the optimal geometries of steel structures, focusing on the height of the conoidal roof and the shape and arrangement of columns. The subsequent analysis included static and strength calculations, dimensioning, and evaluating structural responses to exceptional loading, incorporating novel impact scenarios. The analysis yielded several key insights into the structural efficiency, dynamic behavior, and design optimization of the shelters. The research revealed that both roof geometry and column shape and arrangement significantly influenced material consumption and design effectiveness. The findings indicated that shelters with four straight, vertical, non-corner columns exhibited the most favorable dynamic behavior and highest impact resistance. These shelters also facilitated easy parking for both single-module and double-module roof types. The research findings provide a foundation for the parametric design of functional and structurally resilient parking shelters that cater to urban transportation needs and ecological objectives.

## 1. Introduction

Modern cities grapple with numerous challenges in managing public spaces effectively, such as excessive transportation demands and insufficient green areas. As a result, the significance of creating public spaces that meet both social and environmental needs is increasingly emphasized in various studies [1,2,3]. Rapid urbanization, the growing number of vehicles, and the necessity to adapt to climate change drive the search for sustainable urban space solutions. One promising approach is the effective design of small-scale architecture, such as parking and bike shelters, which, when integrated with green infrastructure, can significantly improve residents’ quality of life. These shelters are becoming crucial in shaping organized and aesthetically pleasing urban environments [4,5]. The potential of shelters equipped with photovoltaic installations and their hybrid functions—utility, environmental, and aesthetic—is increasingly recognized [6,7]. Examples from European cities, such as Copenhagen, Amsterdam, and Rotterdam, illustrate that car parks with vegetated or green roofs are becoming a standard in modern, sustainable urban spaces [8,9]. These solutions are not only prevalent in large cities but are also increasingly common in medium-sized cities, where car parks are incorporated into pocket parks or revitalized public squares [10,11,12]. Furthermore, the development of the “woonerf” concept—streets designed to be pedestrian- and cyclist-friendly, integrating car shelters, bicycle shelters, and greenery—exemplifies a cohesive urban design approach [13]. As noted by Gehl, the quality of life for residents largely depends on the design of everyday spaces, especially transitional areas, like car parks and pavements [14,15,16,17].

From an architectural and material engineering perspective, parking shelters can feature roofs of various shapes, such as flat or curved, and can be covered with different materials, like steel, glass, or polycarbonate. These shelters can be constructed using various materials, including steel, aluminum, and wood. However, steel is most commonly proposed as a structural material for shelters. Given steel’s widespread use in construction and its significant impact on the carbon footprint, optimizing steel structures at the early design stage is crucial.

Steel structures, valued for their lightness and ease of assembly, must comply with specific design standards [18,19,20] and consider both operational conditions and accidental scenarios [21]. Parking shelters, in particular, are subjected to various loads, including exceptional ones like vehicle impacts, which can affect their stability and durability. This vulnerability arises from their function and often limited maneuvering space for parked vehicles. Collisions can be caused by various factors, such as poor driving skills, a failure to maintain a safe distance from objects, excessive speed, and adverse environmental conditions. Most scientific publications on car collisions primarily focus on incidents involving two vehicles or collisions with steel or reinforced concrete barriers along roads. Study [22] suggests enhancing vehicle collision simulations using finite element analysis to examine the interactions between reinforced concrete barriers and bridge decks. Various numerical analysis techniques and experimental methods are used to evaluate the performance of reinforced concrete barriers [23,24] and the performance of hybrid roadside barriers developed using waste materials [25]. In [26], a numerical model of a vehicle–guardrail collision is built, and a novel style for an assembled rolling guardrail is presented. Moreover, the laboratory impact tests have been increasingly adopted for evaluating the road safety performance of barrier systems subjected to vehicle collisions [27]. On the other hand, ref. [28] indicates that vehicle collisions with buildings occur mainly in urban areas, and statistical data on the frequency of these incidents are provided. Study [29] examines the impact of a vehicle collision on the stability of a portal frame, a key structural component of a steel hall. Specifically, it assumes a scenario where a 20,000 kg heavy-duty delivery truck collides with a main support column, with the impact direction being perpendicular to the hall’s side wall. The research focuses on the dynamic effects of such impacts while disregarding the nonlinear behavior of the material. The findings indicate that at low vehicle speeds, the stability of the structure mitigates the dominance of the impact.

In the case of parking shelters, the columns supporting the roof are the structural elements most exposed to collisions. Given the frequency of such incidents, analyzing the impact of vehicle collisions on parking shelter columns is crucial. The additional force acting on the columns during collisions can cause damage and the loss of structural stability. Therefore, this research addresses the problem of shaping effective steel parking shelters that are resistant to collisions with parking vehicles. In particular, it examines the influence of the roof shape and the shapes and positions of columns on the shelters’ effectiveness.

Unlike previous methods [29], genetic algorithms and optimization are employed at the outset to determine the optimal geometries of the shelter structures and the best shapes and arrangements of columns. While the analysis of the impact of additional forces acting on columns during a collision is similar to the previous study [29], this research focuses on collisions involving passenger cars, rather than trucks. Additionally, unlike previous studies [29], this research considers two different directions of dynamic force impacts due to potential collision scenarios. This approach allows for the examination of various shelters with two types of roofs and different column configurations, enabling the selection of optimal solutions. The obtained results can serve as a foundation for the rational design of parking shelters.

## 2. Materials and Methods

**Figure 1:**
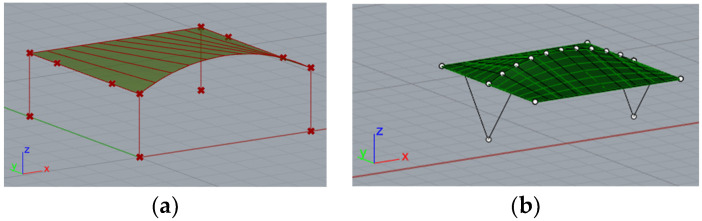
Examples of roof structures: (**a**) single-module (type A structure); (**b**) double-module (type B structure).

Parking shelters with curved roofs were designed parametrically using Rhinoceros 3D (https://www.rhino3d.com/ (accessed on 27 April 2025)) [30], a software extensively utilized in architecture and engineering for creating intricate shapes and forms. Rhinoceros 3D, enhanced by various plug-ins, provides a wide range of functions. In this research, plug-ins such as Grasshopper and Karamba 3D (https://karamba3d.com/ (accessed on 5 May 2025)) were employed [31]. Grasshopper, a popular plug-in, facilitates visual programming through logically connected components, enabling rapid parameter adjustments, the creation of multiple design variants, and genetic optimization. While Grasshopper is used for shaping geometry, Karamba 3D is utilized for structural analysis. Previous publications have demonstrated the shaping of various roof structures using genetic algorithms and considering various shaping criteria [32,33,34,35]. In the presented research, the geometry of the car shelters features a conoid roof supported by columns. Each roof structure includes two ridges, one curved and one straight, positioned in vertical and parallel planes that form the directrices of the conoid roof, along with straight purlins in vertical planes perpendicular to the ridges’ planes, Figure 1. The roof of the structure can be attached to columns of various shapes: straight, V-shaped, and Y-shaped. Additionally, both single-module and double-module roof structures, consisting of two conoid segments, can be considered. In a further analysis, they will be referred to as type A structures and type B structures, respectively, as shown in Figure 1.

The research was divided into three main stages. The first stage focused on optimizing the geometry of the car park structures using Rhinoceros 3D with Grasshopper and Karamba 3D, primarily concerning the optimization of the column shapes and arrangements. The second stage included static-strength calculations and the dimensioning of individual structural elements using Autodesk Robot Structural Analysis Professional for the optimized geometric forms of the car shelters obtained through Karamba 3D. This stage also analyzed the structure’s behavior in an accidental scenario when a vehicle hit a column supporting the shelter roof. The final stage consisted of a comparative analysis of the optimization results, their evaluation, the selection of the most efficient structures, column arrangement, and drawing conclusions to support effective car shelter design.

## 3. Results

### 3.1. Shaping Geometry of Steel Parking Shelter Structures and Determining the Arrangement of Columns

**Figure 2:**
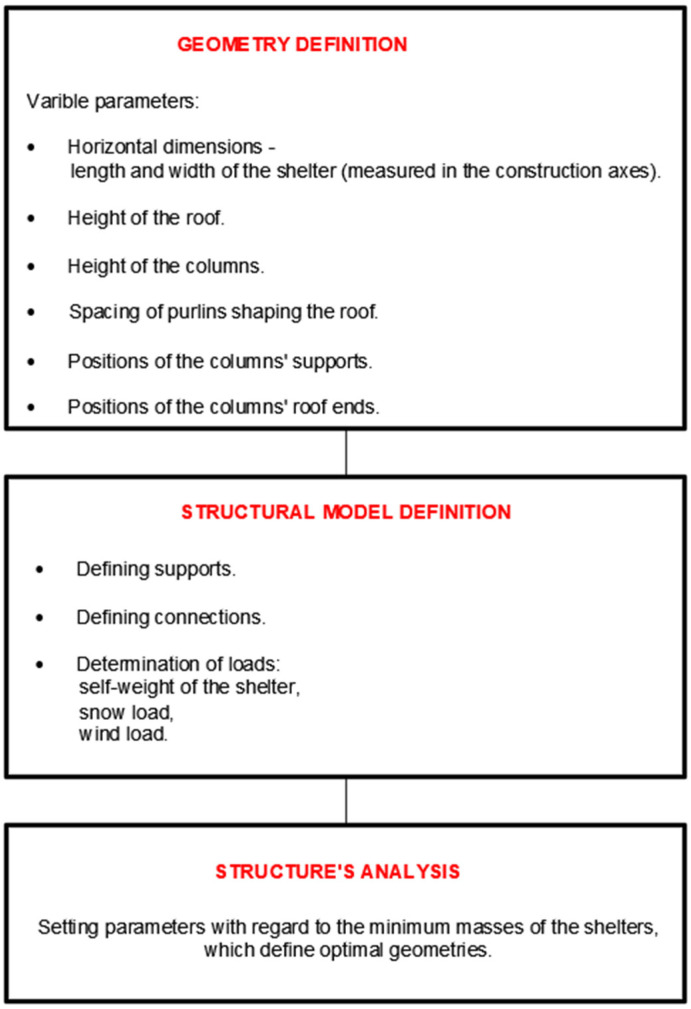
A general scheme of the geometry and structural model definitions.

In Rhinoceros 3D software, a block script was created using a visual programming plug-in, Grasshopper, to describe the geometry of shelter structures in a parametric manner. The geometry was defined using components connected by wires enabling the flow of information between them. Each car shelter was composed of a curved roof structure supported by columns. After some modifications, the script allowed for the creation of shelters with single-module or double-module roofs, Figure 1. The general scheme for creating geometry and defining the structural model is shown in Figure 2. The variable parameters used in the script included the horizontal dimensions, roof height, column height, the spacing of the purlins shaping the roof, the positions of the column supports, the positions of the column-to-roof connections, and the position of the central joint of the column in the case of Y-shaped columns. After creating the geometric part of the script using Grasshopper, the script was extended with Karamba 3D, a structural analysis plug-in. The supports, loads, materials, and connections of the structures were determined using appropriate components, followed by preliminary static calculations using first-order linear analyses.

**Figure 3:**
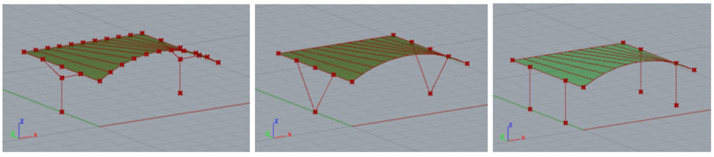
Basic structures subjected to analysis.

The construction material used was S235 steel, which is a very popular type of structural steel extensively utilized in construction. However, round and rectangular profiles were adopted for the structural elements. Rigid connections were employed throughout. The basic structural shapes analyzed are presented in Figure 3.

**Figure 4:**
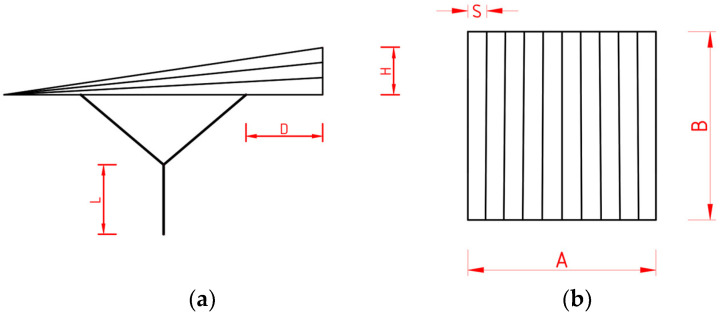
Views of the structure: (**a**) side view; (**b**) top view.

To optimize the structures and select the most effective geometry, it was initially assumed that each parking shelter had horizontal dimensions, A and B, equal to 6 m, and the vertical distance from the ground to the edge purlin was 2.1 m, Figure 4. Furthermore, it was assumed that the total height, H, of the roof varied within the range of 0.8 to 1.0 m, Figure 4. The distance, D, of the upper end of the column from the end of the ridge ranged from zero to one-third of the edge purlin’s length, which was 1.5 m. However, the length, L, of the main part of the column for Y-shaped columns ranged from 0 to 1 m. Each roof module was divided by purlins into 10 parts equal to S from the top view, Figure 4. The ranges of the adopted parameters were established on the basis of engineering practice and ensuring the aesthetic appearance of the structures.

The analysis incorporated a permanent vertical load representing the structure’s self-weight and polycarbonate roof covering. Additionally, a vertical snow load was applied globally to the roof, and a wind load was applied locally (perpendicular to the roof surface). Two cases of a snow load on the roof (symmetrically distributed and asymmetrically distributed) and two cases of a wind load (the load from above and below) were considered. The environmental loads were specified for Tarnobrzeg (Poland), located in wind zone 2, with a basic velocity pressure of 0.30 kN/m^2^, and located in snow zone 2, with a ground snow load of 0.9 kN/m^2^. Using Karamba 3D, it was possible to integrate the definition of a parametric shelter model with finite element calculations and an optimization algorithm to explore various shapes. Consequently, several genetic optimizations were conducted for various load case scenarios using the Galapagos evolutionary solver, focusing on optimizing the cross-sections. The primary objective of each optimization was to minimize the mass of the structure. As a result, the simulation identified the geometry with the lowest mass that met the specified criteria (established parameter ranges).

The results of the simulations gave the values of parameters: H, D, and L, as follows:

- H = 0.8 m;
- D = 1.5 m;
- L = 1.5 m.

These parameters determined the optimal shelter geometries for the assumed initial criteria (ranges of parameters). The same parameter values were used for both structures of type A and structures of type B.

### 3.2. Detailed Static Calculation and Optimization

**Figure 5:**
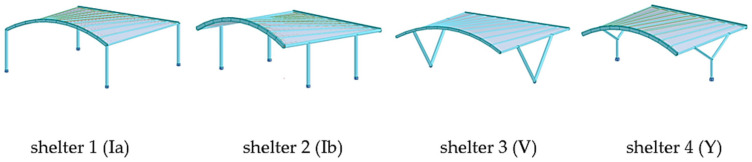
The shelters 1, 2, 3, and 4 with roof type A and with columns of types Ia, Ib, V, and Y, respectively.

**Figure 6:**
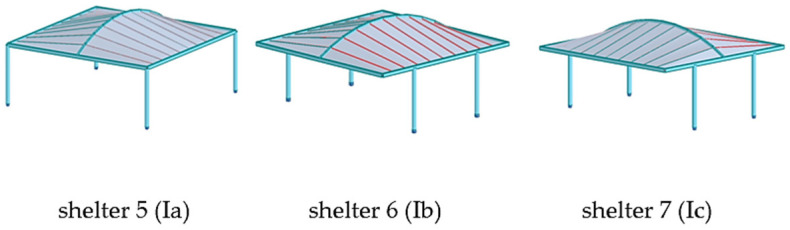
The shelters 5, 6, and 7 with roof type B and with columns of type Ia, Ib, and Ic, respectively.

**Figure 7:**
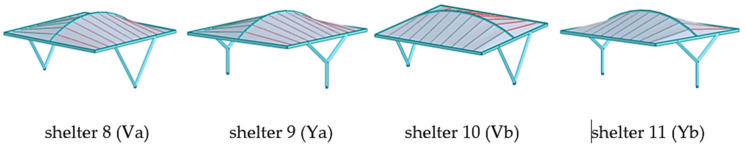
The shelters with roof type B and with columns of type Va, Ya, Vb, and Yb, respectively.

Due to the fact that the static analysis carried out using Karamba 3D should be considered as simplified and approximate, the structural models obtained as a result of simulations performed by means of Karamba 3D were next subjected to detailed static and strength analyses using Autodesk Robot Structural Analysis Professional. The types of shelter models analyzed are presented in Figure 5, Figure 6 and Figure 7.

Under standard design conditions, to verify the ultimate limit state (ULS), the following characteristic loads and parameters were considered: the self-weight of the steel structure and the polycarbonate roof covering, as well as environmental loads, such as wind and snow. Two cases of snow loads (uniformly distributed and non-uniformly distributed) were considered, while the wind load was generated automatically, taking into account the symmetry of the structure. Additionally, for accidental design situations, a load resulting from a vehicle impact on a shelter column was considered. However, further details regarding this load are provided in a subsequent section. Load combinations were developed in accordance with relevant standards. A structural analysis was performed using Autodesk Robot Structural Analysis Professional 2024, and the structural members were optimized for mass. The analysis process was divided into two main stages. The first stage involved verifying the stability of the structural elements under permanent design loads. The second stage addressed their stability under accidental conditions, specifically the impact of a vehicle on a selected column. For the permanent design situation, a first-order linear static analysis was used to assess the structural behavior. The global stability of the designed parking shelters was evaluated, and strength calculations were carried out, assuming a buckling length factor equal to two for columns and equal to one for the rest of the structural members. The buckling length factor was assumed to be the same for all the columns because they were similarly attached to the structure and the ground and they were made of similar profiles and steel. The cross-sections of the elements were optimized using both circular and rectangular hollow profiles. The adoption of such profiles was dictated by technological reasons. However, the dimensions of the profiles obtained are presented in Table 1 and Table 2.

### 3.3. Structural Stability Assessment Under Accidental Load Conditions

The second stage of the analysis focused on assessing the structural performance in the case of a vehicle collision. This evaluation followed the procedures outlined in Annex C—dynamic design considering an impact [24]. The magnitude of the dynamic load resulting from the impact, along with the applied interaction model, was determined based on the provided guidance. An impact event involves the interaction between a moving vehicle and a structural component when the vehicle’s kinetic energy is rapidly converted into deformation energy within the structure. To calculate the forces generated by this dynamic interaction, the mechanical properties of both the impacting vehicle and the structural elements were considered.

Designing structures for dynamic loads often involves using equivalent static forces. More detailed analyses may also account for additional factors, such as the effects of dynamic loading over time and material nonlinearity. The impacts in dynamic analyses are typically classified into two categories: hard impact, when most of the energy is absorbed by the vehicle and the structure is treated as rigid; and soft impact, when the structure is designed to deform, allowing it to absorb energy through displacement. In the analysis conducted, a hard-impact scenario was assumed as no deformation of the structure during impact was allowed. This means that the dynamic effects of the collision were considered, while nonlinear material behavior was not included. This choice was made to represent a conservative, worst-case scenario, in which the majority of the kinetic energy of the vehicle is rapidly transferred to the structural element, assuming the minimal energy absorption or deformation of the impacting vehicle itself. In hard-impact models, the structure is typically treated as elastic, and the force–time history is characterized by short-duration, high-intensity impulses. This contrasts with soft-impact models, where a significant portion of the impact energy is dissipated through the plastic deformation of the structural element or the vehicle, resulting in longer impact durations and lower peak forces.

The decision to adopt a hard-impact model in this study was driven by two key considerations. First, it provides an upper-bound estimate of the forces that the structure might experience during an accidental collision, ensuring that the design remains safe, even under extreme conditions. Second, hard-impact conditions are more critical for slender structural components, such as the columns of parking shelters, where sudden, concentrated loading poses a greater risk of local failure or instability.

#### Dynamic Design for Impact Calculation—Method Description

The first step was calculating the dynamic interaction force using Formula (1) [24]:

F_DYN_ = v_r_ × √(k × m) (1)

where

v_r_—object velocity during collision,
k—equivalent elastic stiffness,
m—impacting object mass.

**Figure 8:**
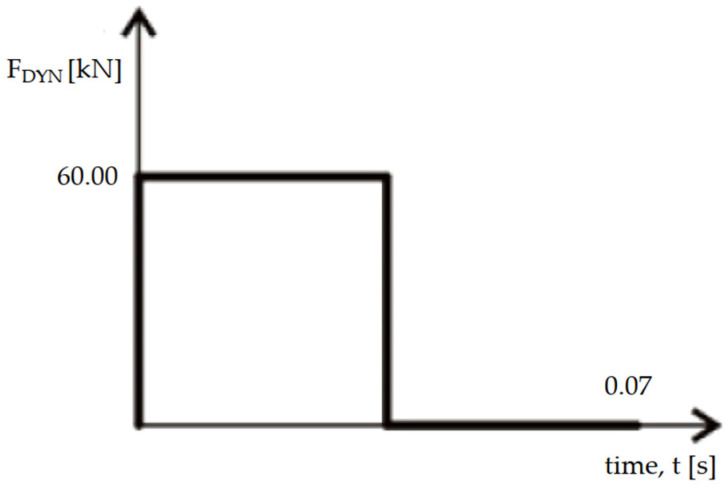
The dynamic impulse from a vehicle impact.

The impact force was represented as a rectangular impulse applied over the surface of the structure. The duration of the impulse in the equation was established using Formula C.2 [21]. The dynamic impulse from a vehicle impact is presented graphically in Figure 8.

The point of force was established as 0.50 m above the ground, as experienced in passenger cars. Equation (1), from the initial phase, was utilized to determine the peak dynamic forces acting on the structure’s exterior surface. These forces could potentially amplify the effects of dynamic loading. To estimate the upper limit of these forces, it was assumed that the structural response remained purely elastic. The applied load was modeled as a step function, instantly reaching its peak value and remaining constant over time.

In the analyzed scenario, the following conditions were assumed:

- A passenger vehicle with a mass of m = 1500 kg impacted a structural column;
- The incident occurred within the maneuvering zone adjacent to the shelter;
- The vehicle’s speed was 10 km/h (2.78 m/s);
- The impact was perpendicular to the shelter’s column;
- The vehicle’s equivalent elastic stiffness was k = 300 kN/m.

The assumed vehicle speed of 10 km/h was selected to reflect a realistic scenario of maneuvering within a parking facility. Such a speed is representative of typical vehicle movements during parking operations, including entering or exiting parking spaces and navigating narrow maneuver zones adjacent to shelters. The studies of parking lot dynamics and accident reports indicate that low-speed collisions, generally below 15 km/h, are the most common in such environments. Therefore, adopting a speed of 10 km/h ensures that the analysis corresponds to plausible conditions while maintaining a degree of safety to account for driver error or unexpected acceleration. Including this speed in the analysis supports the relevance and applicability of the findings to real-world parking infrastructure design.

In this conducted analysis, a dynamic force of approximately 60 kN was calculated using Equation (1), while a corresponding static force of 50 kN was obtained from Table C.2 [21].

**Figure 9:**
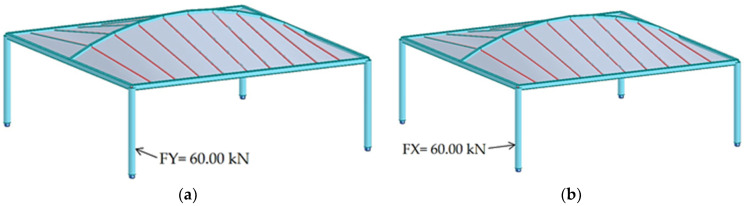
The method of applying the force to the column: (**a**) a scenario of a parking vehicle; (**b**) a scenario of a maneuvering vehicle.

These forces were applied perpendicularly to the column in two distinct configurations, Figure 9. The first configuration simulated a scenario of a vehicle parking. The second configuration assumed the force applied from the side of the column, representing an impact occurring during vehicle maneuvering. The methods of force application are illustrated in Figure 9a,b.

### 3.4. Comparison of Permanent and Accidental Load Scenarios

The structural analysis included the optimization of the members with respect to their masses, taking into account the impact forces resulting from the vehicle collision. The optimization process involved selecting the structural profiles that are listed in Table 3 and Table 4.

The optimization process focused exclusively on load combinations related to the accidental scenario, specifically evaluating the effects of a vehicle impact. As shown in Table 1, Table 2, Table 3 and Table 4, an increase in the column cross-sectional dimensions was observed, reflecting the greater structural requirements needed to ensure stability under such extraordinary conditions. As a result of the optimization, cross-sections of structures with similar utilizations were obtained, Table 5. However, the displacement values of the upper column node impacts were different for various structures. However, they did not exceed the permissible value, Table 5.

Furthermore, a comparison of the internal forces within the structural elements under both permanent and accidental loading conditions was performed. The results of this comparison are provided in Table 6 and Table 7. The dominant load effects under the permanent design situation were identified for a representative column of each shelter. Subsequently, the computed impact force was applied in accordance with the configuration illustrated in Figure 9, and the resulting internal forces corresponding to the accidental design situation were determined.

Table 6 and Table 7 present a comparison of the internal forces in the columns for two roof types (A and B) under permanent and accidental situations. Table 6 shows the results for shelters with roof type A, while Table 7 includes the results for shelters with roof type B. The tables list the bending moments (My, Ed) and the axial forces (NEd), as well as how these forces vary between the loading scenarios. Notable differences, including sign changes in the bending moments, indicate the structural response and sensitivity of different roof configurations under accidental conditions.

Table 8 and Table 9 summarize the results of the time analysis for the shelters with roof types A and B, respectively. The tables include key dynamic parameters: natural frequency, vibration period, and pulsation. Table 8 presents the data for shelters 1 to 4 (roof type A), while Table 9 covers shelters 5 to 11 (roof type B). The results indicate that type B roof structures generally exhibit a wider range of dynamic responses, with some shelters showing significantly higher frequencies and others much lower, suggesting differences in the stiffness and the mass distribution between the two roof types.

## 4. Discussion

Based on the conducted analysis of eleven shelter models designed with two different roof types under both permanent and accidental loading conditions, several key conclusions can be drawn regarding their structural efficiency, dynamic behavior, and design optimization.

The comparison of structural weights across the models indicates that roof geometry plays a crucial role in material consumption and design effectiveness, Table 10 and Table 11. The shelters designed with roof type A consistently exhibit higher structural masses, with permanent situation values ranging between 731 kg and 1002 kg, while roof type B shelters are notably lighter, with masses ranging between 619 kg and 679 kg. These differences persist under accidental situations, reinforcing the finding that type B roofs offer a more material-efficient solution without compromising structural integrity. It is worth noting that, considering similar cross-section utilization, Table 5, the mass of the structure can be regarded as an indicator of its efficiency.

This weight disparity, while directly tied to geometry, also reflects the different force distributions experienced by each configuration. As demonstrated in Table 6 and Table 7, accidental situations cause significant deviations in internal forces—most notably increases in bending moments and, in some cases, the inversion or reduction of axial forces. These structural shifts confirm the hypothesis that accidental scenarios—such as vehicle impacts—demand a more robust lateral resistance strategy, especially for columns.

Moreover, the time analysis results from Table 8 and Table 9 further support this differentiation. Roof type B shelters tend to exhibit higher fundamental frequencies and shorter periods, indicating greater stiffness and better dynamic performance, which are critical in resisting sudden lateral loads. This suggests that roof type B is not only more efficient in terms of weight but also more responsive dynamically.

The optimization of cross-sections, presented in Table 1, Table 2, Table 3 and Table 4, reveals a consistent trend: structural members under accident loading conditions require increased dimensions, particularly for columns and arches. This increase highlights the need for additional stiffness and material strength to prevent instability during exceptional events, aligning with the outcomes of prior research, which advocates for cautious, safety-focused design in the presence of impact loads.

Notably, shelters 2 and 5 exhibited both increased axial forces and elevated bending moments, a compounded loading condition that emphasizes the complexity of real-world accidental events. These cases suggest that design strategies must move beyond isolated axial or bending capacity checks and embrace a more holistic assessment, incorporating combined load effects, dynamic responses, and failure mode considerations.

However, by analyzing Table 10 and Table 11, it can be concluded that shelter 2 (Ib), from the group of structures of type A, and shelter 7 (Ic), from the group of structures of type B, have the smallest masses. For these representative structures, graphs of the time-dependent behavior of the bending moment were prepared.

**Figure 10:**
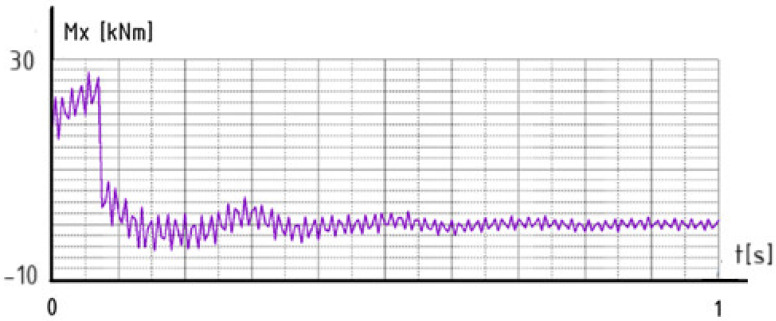
The time-dependent behavior of the bending moment, Mx—shelter 2.

**Figure 11:**
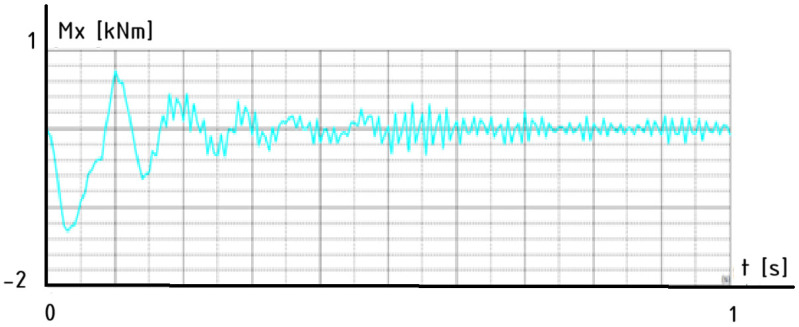
The time-dependent behavior of the bending moment, Mx—shelter 7.

These graphs illustrate the time-domain response of the moment, Mx, at a lower node of the column subjected to an impact from a vehicle, Figure 10 and Figure 11. The first graph corresponds to a structure with a roof type A configuration, Figure 10, while the second graph represents the response for roof type B under the same impact conditions, Figure 11.

These results capture the dynamic behavior of the column immediately following the collision, providing insights into how each roof design influences the structural response, energy dissipation, and stabilization characteristics of the system. The comparison helps evaluate which configuration offers better performance in mitigating dynamic effects resulting from sudden impact loads.

The structural response of the column with roof type A demonstrates effective dynamic behavior under a vehicle impact. The moment, Mx, initially shows high-amplitude oscillations, which rapidly decay due to strong damping. This indicates that the structure is capable of efficiently dissipating the energy transferred from the collision. The system stabilizes quickly, reaching a steady-state with minimal residual vibrations. This performance suggests that shelter 2 (roof type A) contributes positively to the overall rigidity and energy absorption capacity of the column, ensuring a faster return to equilibrium and reduced dynamic stress on the structure.

The moment, Mx, response for the column of shelter 7 (roof type B) reveals a more complex and less stable dynamic behavior. The initial oscillations are irregular and persist for longer, with slower damping and more pronounced fluctuations over time. Although the system eventually stabilizes, the extended transition period and the residual noise suggest that shelter 7 (roof type B) offers a lower damping efficiency or introduces additional flexibility into the structure. As a result, this configuration is less effective in managing impact-induced dynamic loads, potentially leading to greater stress and longer recovery times in real collision scenarios.

## 5. Conclusions

This article presents research findings on the application of genetic algorithms to the design of efficient parking shelter structures. It demonstrates that genetic optimization is a powerful engineering tool for swiftly selecting optimal design variants during the early stages of development. These variants can subsequently undergo various analyses, such as vehicle impact assessments. The study highlights that the geometry of the roof, along with the arrangement and shape of columns, significantly affects both the material efficiency and structural resilience of parking shelters. The findings clearly indicate that structures with type B roofs, characterized by double-module configurations, provide superior material efficiency while offering a favorable dynamic performance, particularly under accidental loading conditions. This suggests that future design choices should prioritize roof geometries like type B, which ensure both a lightweight construction and high stiffness. These characteristics contribute to better energy dissipation and the effective stabilization of the structure after impact events. Additionally, the analysis confirmed that shelters equipped with straight, vertical columns, but ones that are not placed at the corners of the roof, are the most economical, while also allowing for easy parking. Such insights are critical for urban planners and engineers aiming to design structures that are not only functional and economical but also resilient to accidental actions that may occur in dense urban environments.

Looking ahead, further research should involve advanced simulations, accounting for strain-rate effects, plasticity, and progressive failure mechanisms. Such enhancements would yield more accurate cross-section designs, potentially reducing unnecessary material use while preserving safety. The experimental validation of these computational models, particularly for shelters identified as worst-case scenarios (e.g., shelters 4 and 5), would provide essential data for improving design codes and engineering practice.

In conclusion, the findings underline the structural implications of accidental loading and advocate for the early-stage integration of impact-resistant strategies. The demonstrated benefits of lighter, dynamically stiffer roof configurations (such as type B) provide a compelling case for performance-based design in shelter systems, aimed at achieving resilience with optimized resource use.

Moreover, the results highlight the necessity of integrating impact-resistant strategies at the conceptual design stage, as this approach can substantially reduce the need for subsequent reinforcement or retrofitting. The demonstrated benefits of applying parametric and algorithmic design methods also underscore the potential of these tools to facilitate the development of innovative forms that balance architectural aesthetics with high structural performance. This holistic approach can contribute to the creation of functional urban infrastructure aligned with sustainable development objectives.

In summary, the presented research provides certain guidance for the future of parking shelter design, offering principles and methodologies to achieve resilient, efficient, and sustainable structures adapted to the challenges of modern urban spaces.

## Figures and Tables

**Table 1:** Optimized cross-section results for the permanent design situation. Roof type A.

Column Type	Shelter Number	Column’s Cross-Section [mm × mm]	Arch 1 Cross-Section [mm × mm]	Edge Beam Cross-Section [mm × mm]	Edge Purlin Cross-Section [mm × mm]	Purlin Cross-Section [mm × mm]
Ia	shelter 1	RO 139.7 × 4.5	RP 140 × 80 × 4	RP 140 × 80 × 4	RO 88.9 × 3.2	RO 88.9 × 3.2
Ib	shelter 2	RO 168.3 × 5.0	RP 140 × 80 × 4	RP 180 × 100 × 4	RO 159.0 × 4.5	RO 88.9 × 3.2
V	shelter 3	RO 127.0 × 4.0	RP 180 × 100 × 4	RP 200 × 100 × 5	RO 168.3 × 4.5	RO 88.9 × 3.2
Y	shelter 4	RO 139.7 × 4.5	RP 200 × 100 × 4	RP 200 × 100 × 5	RO 168.3 × 4.5	RO 88.9 × 3.2

**Table 2:** Optimized cross-section results for the permanent design situation. Roof type B.

Column Type	Shelter Number	Column’s Cross-Section [mm × mm]	Arch 1 Cross-Section [mm × mm]	Edge Beam Cross-Section [mm × mm]	Edge Purlin Cross-Section [mm × mm]	Purlin Cross-Section [mm × mm]
Ia	shelter 5	RO 127.0 × 4.0	RP 120 × 80 × 4	RP 120 × 80 × 4	RP 120 × 80 × 4	RO 54.0 × 3.2
Ib	shelter 6	RO 101.6 × 3.6	RP 120 × 80 × 4	RP 120 × 80 × 4	RP 120 × 80 × 4	RO 54.0 × 3.2
Ic	shelter 7	RO 101.6 × 3.2	RP 120 × 80 × 4	RP 120 × 80 × 4	RP 120 × 80 × 4	RO 48.3 × 3.2
Va	shelter 8	RO 114.3 × 3.2	RP 120 × 80 × 4	RP 120 × 80 × 4	RP 120 × 80 × 4	RO 48.3 × 3.2
Ya	shelter 9	RO 133.0 × 4.0	RP 120 × 80 × 4	RP 120 × 80 × 4	RP 120 × 80 × 4	RO 48.3 × 3.2
Vb	shelter 10	RO 114.3 × 3.2	RP 120 × 80 × 4	RP 120 × 80 × 4	RP 120 × 80 × 4	RO 48.3 × 3.2
Yb	shelter 11	RO 101.6 × 3.6	RP 120 × 80 × 4	RP 140 × 80 × 4	RP 140 × 80 × 4	RO 48.3 × 3.2

**Table 3:** Optimized cross-section results for the accident situation. Roof type A.

Column Type	Shelter Number	Column’s Cross-Section [mm × mm]	Arch 1 Cross-Section [mm × mm]	Edge Beam Cross-Section [mm × mm]	Edge Purlin Cross-Section [mm × mm]	Purlin Cross-Section [mm × mm]
Ia	shelter 1	RO 159.0 × 4.5	RP 140 × 80 × 4	RP 140 × 80 × 4	RO 88.9 × 3.2	RO 88.9 × 3.2
Ib	shelter 2	RO 168.3 × 5.0	RP 140 × 80 × 4	RP 180 × 100 × 4	RO 159.0 × 4.5	RO 88.9 × 3.2
V	shelter 3	RO 159.0 × 4.5	RP 180 × 100 × 4	RP 200 × 100 × 5	RO 168.3 × 4.5	RO 88.9 × 3.2
Y	shelter 4	RO 168.3 × 4.5	RP 200 × 100 × 4	RP 200 × 100 × 5	RO 168.3 × 4.5	RO 88.9 × 3.2

**Table 4:** Optimized cross-section results for the accident situation. Roof type B.

Column Type	Shelter Number	Column’s Cross-Section [mm × mm]	Arch 1 Cross-Section [mm × mm]	Edge Beam Cross-Section [mm × mm]	Edge Purlin Cross-Section [mm × mm]	Purlin Cross-Section [mm × mm]
Ia	shelter 5	RO 159 × 4.5	RP 120 × 80 × 4	RP 120 × 80 × 4	RP 120 × 80 × 4	RO 54.0 × 3.2
Ib	shelter 6	RO 159 × 4.5	RP 120 × 80 × 4	RP 120 × 80 × 4	RP 120 × 80 × 4	RO 54.0 × 3.2
Ic	shelter 7	RO 159 × 4.5	RP 120 × 80 × 4	RP 120 × 80 × 4	RP 120 × 80 × 4	RO 48.3 × 3.2
Va	shelter 8	RO 159 × 4.5	RP 120 × 80 × 4	RP 120 × 80 × 4	RP 120 × 80 × 4	RO 48.3 × 3.2
Ya	shelter 9	RO 168.3 × 4.5	RP 120 × 80 × 4	RP 120 × 80 × 4	RP 120 × 80 × 4	RO 48.3 × 3.2
Vb	shelter 10	RO 159 × 4.5	RP 120 × 80 × 4	RP 120 × 80 × 4	RP 120 × 80 × 4	RO 48.3 × 3.2
Yb	shelter 11	RO 159 × 4.5	RP 120 × 80 × 4	RP 140 × 80 × 4	RP 140 × 80 × 4	RO 48.3 × 3.2

**Table 5:** Column cross-section utilization for both permanent and accidental situations, max node displacement.

Column Type	Shelter Number	Permanent Situation Column Cross-Section Utilization [%]	Accident Situation Column Cross-Section Utilization [%]	Max Node’s Displacement [cm]
Ia	shelter 1	0.91	0.93	1.20
Ib	shelter 2	0.94	0.92	1.30
V	shelter 3	0.90	0.88	1.20
Y	shelter 4	0.98	0.98	1.40
Ia	shelter 5	0.80	0.87	0.50
Ib	shelter 6	0.98	0.89	1.00
Ic	shelter 7	0.95	0.90	0.30
Va	shelter 8	0.98	0.90	1.00
Ya	shelter 9	0.92	0.98	0.70
Vb	shelter 10	0.98	0.91	0.80
Yb	shelter 11	0.97	0.93	1.30

**Table 6:** Force comparison for permanent and accident situations. Roof type A.

Column Type	Shelter Number	Columns—Permanent Situation		Columns—Accident Situation	
		My, Ed	Ned	My, Ed	Ned
		[kNm]	[kN]	[kNm]	[kN]
Ia	shelter 1	17.44	19.87	23.84	0.05
Ib	shelter 2	23.82	20.69	23.22	0.43
V	shelter 3	−0.14	25.84	19.82	35.28
Y	shelter 4	0.63	42.18	−27.94	0.00

**Table 7:** Force comparison for permanent and accident situations. Roof type B.

Column Type	Shelter Number	Columns—Permanent Situation		Columns—Accident Situation	
		My, Ed	Ned	My, Ed	Ned
		[kNm]	[kN]	[kNm]	[kN]
Ia	shelter 5	−3.79	16.35	−22.08	0.15
Ib	shelter 6	2.85	16.23	−22.39	−0.97
Ic	shelter 7	5.28	16.23	−22.67	0.16
Va	shelter 8	−1.96	17.73	19.07	34.89
Ya	shelter 9	1.05	26.00	−27.30	0.00
Vb	shelter 10	−2.67	18.06	−0.75	−0.18
Yb	shelter 11	−1.62	26.26	−23.38	−0.52

**Table 8:** Time analysis parameter results. Roof type A.

Column Type	Shelter Number	Frequency [Hz]	Period [s]	Pulsation [1/s]
Ia	shelter 1	4.64	0.22	29.18
Ib	shelter 2	3.20	0.31	20.08
V	shelter 3	2.42	0.41	15.28
Y	shelter 4	0.52	1.93	3.25

**Table 9:** Time analysis parameter results. Roof type B.

Column Type	Shelter Number	Frequency [Hz]	Period [s]	Pulsation [1/s]
Ia	shelter 5	6.20	0.16	38.98
Ib	shelter 6	2.13	0.47	13.37
Ic	shelter 7	2.27	0.44	14.25
Va	shelter 8	2.52	0.40	15.86
Ya	shelter 9	1.07	0.93	6.74
Vb	shelter 10	2.71	0.37	17.03
Yb	shelter 11	1.33	0.75	8.35

**Table 10:** Summary of weights for shelters in permanent and accidental situations. Type roof A.

Column Type	Shelter Number	Permanent Situation Shelter Mass [kg]	Accident Situation Shelter Mass [kg]
Ia	Shelter 1	731	747
Ib	Shelter 2	941	941
V	Shelter 3	949	999
Y	Shelter 4	1002	1032

**Table 11:** Summary of weights for shelters in permanent and accidental situations. Roof type B.

Column Type	Shelter Number	Permanent Situation Shelter Mass [kg]	Accident Situation Shelter Mass [kg]
Ia	shelter 5	679	694
Ib	shelter 6	652	719
Ic	shelter 7	619	694
Va	shelter 8	645	728
Ya	shelter 9	667	725
Vb	shelter 10	645	728
Yb	shelter 11	669	757

## Data Availability

The original contributions presented in this study are included in the article. Further inquiries can be directed to the corresponding author.
